# Retrosternal Percutaneous Tracheostomy: An Approach for Predictably Impossible Classic Tracheostomy

**DOI:** 10.1155/2010/397270

**Published:** 2010-04-07

**Authors:** Philippe Biderman, Avi A. Weinbroum, Yael Rafaeli, Eyal Raz, Eyal Porat, Ory Wiesel, Oded Szold

**Affiliations:** ^1^Department of Cardiothoracic Surgery, Rabin Medical Center, Beilinson Campus, Petach Tikva 49100, Israel; ^2^ Sackler Faculty of Medicine, Tel Aviv University, Ramat Aviv 69978, Israel; ^3^Post-Anesthesia Care Unit, Tel Aviv Sourasky Medical Center, Tel Aviv 64239, Israel; ^4^Department of Anesthesia, Rabin Medical Center, Beilinson Campus, Petach Tikva 49100, Israel; ^5^Surgical Intensive Care Unit and Department of Surgery “B”, Tel Aviv Sourasky Medical Center, Tel Aviv 64239, Israel

## Abstract

Percutaneous tracheostomy is a routine procedure in intensive care units. In cases of very low position of the larynx, cervical spine deformation, morbid obesity, or neck tumor, performance of the classic tracheostomy is inapplicable. Retrosternal approach to tracheostomy in such 20 patients is herein reported. After preoperative neck computerized tomography to define the neck anatomy, a small suprasternal incision followed by a short retrosternal tissue dissection to expose the trachea was done; the trachea was then catheterized at the level of the 2nd ring in the usual tracheostomy manner. The immediate and late (≥6 months) outcomes were similar to that of the standard tracheostomy. Thus, percutaneous retrosternal tracheostomy is safe in patients with abnormal positioning of the trachea or neck constitution. It is a bedside applicable technique, that, however, requires caution to avoid hazardous vascular complications.

## 1. Introduction

Tracheostomy has become a common practice in the ICU, despite of the recent study that failed to demonstrate any major benefit versus prolonged intubation [1]. The previous prospective, randomized studies have shown benefits of early tracheostomy over endotracheal tube (ETT), with regard to reduction of mortality and morbidity and shortening of the duration of mechanical ventilation and ICU stay [2]. The percutaneous tracheostomy (PCT) technique has become very popular in ICUs over the past decade, and several publications have favorably compared its safety to that of the standard open surgical tracheostomy [3, 4]. It is now the procedure of choice for elective tracheostomy in many critically ill adult patients [3]. The recently published guidelines for PCT have further helped to decrease the immediate complication rate [5]. Additional advantages of PCT are the reduction in lag time between intubation and tracheostomy, and cosmetic considerations, all accounting for the popularity of the PCT in the ICU [6].

An open tracheostomy has occasionally been preferred when there are technical difficulties or the need to perform PCT in patients with “difficult” neck anatomy. Until recently, morbid obesity or urgent tracheostomy was considered as being contraindications for PCT, but this was shown to be seldom justifiable [7–9]. Heyrosa et al. demonstrated reduction in the complication rate of PCT by using an adjuvant bronchoscopy, as compared to the open technique [7].

A complex neck anatomy in which the trachea cannot be palpated between the cricoid cartilage and the sternum poses a challenge when tracheostomy is required. Abnormal anatomy also includes pathological flexion of the spine (neuromuscular disease with deformation), fixation of the spine after cervical-spine trauma with a hallow, conditions after cervical-spine surgery for tumor, and morbidly obese patients, or individuals suffering from benign or malignant tumor of the neck. In these latter, splitting of the sternum and or performing thyroidectomy is indicated before the tracheostomy is performed [10].

We herein describe an original and safe technique for performing a modified PCT in patients with pathological neck anatomy or contraindication to open, classic tracheostomy.

## 2. Methods

### 2.1. Retrosternal PCT Technique

Due to the low level at which the tracheal catheterization needed to be carried out in the selected cases, we were concerned about the risk of bleeding due to accidental puncture of or even penetration into vessels, such as the innominate artery or vein. These latter are anatomically positioned just behind and at the sternoclavicular joint level and frequently alter their position in correspondence to the trachea [11]. In such cases, the anatomical relationships of these and the surgical site of the tracheal penetration are troublesome [12, 13]. As such, all of the study patients underwent neck angiographic computerized tomography (CT) in order to better define the overall anatomical relationship within the neck, before the retrosternal procedure was undertaken. The CT also allowed precise measurement of the distance from the skin to the tracheal rings. In one case, because of the medial and high position of the innominate artery, the procedure was not performed and the patient remained intubated with an ETT.

During the procedure, heart rate and blood pressure were monitored continually, as were SaO_2_ and E_T_CO_2_ levels (CardiocapTM, Datex, Helsinki, Finland). Intravenous propofol (40–60 mg) or midazolam (2-3 mg), fentanyl (50–100 *μ*g), rocuronium (30–50 mg) or vecuronium (3–6 mg), and N_2_O/O_2_ enriched with isoflurane as deemed necessary by the anesthesiologist composed the anesthesia. The patients were monitored postoperatively for as long as necessary, based on the simplicity or difficulty of the procedure itself.

The procedures were performed by an intensive care specialist, an assistant (either an ICU physician or a surgeon), and an anesthesiologist in the ICU, or in the OR if another invasive intervention was required. After positioning the patient in order to ease the intervention, and if possible, extending the neck maximally, the anterior side of the neck and the upper part of the chest were scrubbed with polydine solution and alcohol, and then draped. A 2-cm vertical incision of the skin was performed just above the sternal notch. A small curved mosquito clamp was used to dissect the underlying tissue 2-3 cm below and behind the sternal notch, that is, comparable to the first steps of a mediastinoscopy. Throughout the procedure, a fiberoptic bronchoscope was positioned within the ETT, to visualize and verify a correct point of entry into the tracheal lumen of the device [14]. When the trachea was palpated under the sternum through the dissection, the anesthesiologist withdrew the ETT proximally, to the subglottic region, however, still ventilating the patient. The trachea was mostly located 2-3 cm below the sternal notch, usually at a depth of 4–8 cm under the skin. The trachea was punctured with a needle between the first and the second tracheal rings or between the second and the third tracheal rings, guided by the operator's fingertip. The angle of the needle at the skin was between 30–45 degrees. A wire was then introduced into the trachea according to the standard PCT technique. Dilatation was then performed using the “blue rhino” set, and a tracheostomy cannula was introduced (an Adjustable Flange Tracheostomy Tube, Portex, Hythe, Kent, UK, was used in 60% of the cases; the rest utilized the Shiley Cuffed Tracheostomy Tube, Tyco, Pleasanton, Calif, USA). The fiberoptic scope was now advanced through the cannula to confirm its appropriate positioning (the tip lying above the carina, avoiding one-lung migration). It was then connected to the ventilator and commonly sutured to the skin.

## 3. Results

**Figure 1:**
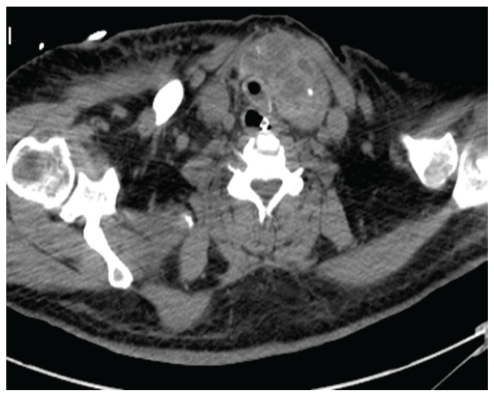
A CT in a patient with a short neck and with a very large goiter. The CT cut is approximately 1 cm above the sternal notch. The trachea is surrounded by the goiter and cannot be safely reached from the anterior portion of the neck, unless thyroidectomy is performed in advance.

**Figure 2:**
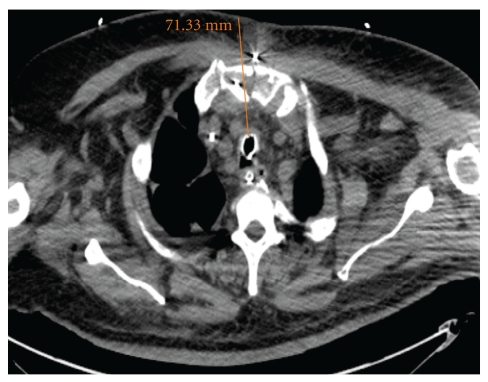
CT of an obese patient. Due to the distance of the trachea from the anterior surface of the neck (>7 cm from the skin), the trachea could be reached reasonably easily and safely from a point under the sternum.

Between 2004–2007 a total of 1,508 patients underwent either open tracheostomy or PCT in our institution, 21 of them were refuted the above and thus scheduled for retrosternal percutaneous tracheotomy (Figures 1 and 2). Retrosternal PCT was withheld in one patient at the last minute, because of the medial and high position of the innominate artery; he remained intubated and ventilated through an ETT. The anatomical landmarks could not be palpated and the cricoid or thyroid cartilages could not be identified in thirteen patients. The trachea could not be reached during an open tracheostomy in the other seven patients due to the position of the first tracheal ring under and behind the sternum. Two patients had more than one anatomical problem that impeded the performance of routine open tracheostomy. The procedure was halted in the above 7 patients who returned to the ward with their ETT. They then underwent CT-angio and based on the results all were referred to retrosternal PCT which was performed uneventfully.

Twelve of the study patients were males and eight were females. The mean age of the study group was 54.5 years (range 21–78), and the average APACHE II score was 25. The observational group had similar values (data not shown). Their anatomical difficulties are detailed in Table 1. Thirteen procedures were performed in the ICU and 7 in the OR; the mean operative time was 35 min (ranges 28–55 min). There were no differences in the procedure outcome whether done in the OR or in the ICU. Neither significant bleeding nor other serious complications occurred perioperatively (Table 2).

Three patients displayed significant but reversible decrease in SaO_2_ (minimum 88% for up to 6 min). These were secondary to bloody secretions in the bronchi, and improved rapidly after suction. There were no cardiac significant changes. The overall mortality during the hospitalization period was 30% (6 patients); none of the fatalities was related to the procedure. This rate has been reported following standard PCTs or the classic (open) technique [2]. All patients were closely followed-up during their stay; the median length of followup was 8 months (range 6–11 months), and the fourteen survivals depicted no long-term complications, including tracheal stenosis secondary to the procedure, during a period of 1 year afterwards. One patient developed moderate tracheomalacia, but did not require surgical repair. Importantly, despite the low puncture site of the trachea, no case of tracheo-innominate fistula was recorded.

## 4. Discussion

We describe a straightforward and safe modified PCT technique that provides a solution to situations where the standard open tracheostomy is impossible, due to neck impediments or anatomical abnormalities, and where the only alternative could be the splitting of the sternum. In ten of our patients, the anatomical landmarks were not palpable prior to surgery and the cricoid or thyroid cartilages were not identifiable. In the other seven patients failure to reach the trachea occurred during an open tracheostomy, due to the abnormal position of the first tracheal ring, under the sternum. In two patients, more than one anatomical problem hampered the performance of the routine tracheostomy. Indeed, the only solution for these patients would have been to split the sternum, a procedure that bears with it long-lasting pain and cosmetic residues, or proceed with a thyroidectomy before the tracheostomy (where applicable). These latter eventualities are performed prior to permanent tracheostomy rather than temporary ones, the former could be complicated by infection and repeated interventions. Importantly, our cohort included five trauma patients, pointing to the fact that this subgroup of patients may also benefit from this technique rather than being ventilated via ETT for a long time.

A “difficult” neck—and upper retrosternal region—anatomy may often pose serious challenges when performing the classic open tracheostomy. Percutaneous tracheostomy (PCT) had initially been preserved for the “easy” cases. After gaining sufficient experience, PCT has now become the procedure of choice for most ICU patients, including “difficult” cases, such as the morbidly obese patients. Nevertheless, this is not the only solution for all patients anymore, especially for ICU cases. Given the presence of vast and major morbidity in these patients, the retrosternal approach of the trachea, the combination of the percutaneous technique and a short dissection under the sternum, seems optimal. The technical setup and the immediate outcome were similar for procedures that were carried out both in the OR or in the ICU, as was for the long-term outcome; these safety indices are analogous to those after the classic tracheostomy, which is encouraging. Importantly, despite the unusual site of the tracheas, none of the patients suffered from subcutaneous emphysema, which is advantageous in post-trauma patients.

Finally, the routine carrying out of angio-CT of the neck and the upper chest prior to the procedure must be emphasized: it prevents potential serious complications, mostly accidental puncture of the great vessels or the lungs. This imaging technique would be essential in obese patients as well, indicating the distance of the trachea from the skin, although the CT gantry in our institution is limited to 150-kg body weight.

In conclusion, although this report encompasses a small portion of the cohort undergoing PCT, restrosternal percutaneous tracheostomy is currently performed in our institution because considered a safe alternative to PCT that unexpectedly turns difficult or impossible to perform, as may occur in morbidly obese patients. This technique may expand one's clinical acumen, providing a reliable solution when the classical open tracheostomy or the PCT are inapplicable due to low position of the larynx. Preoperative neck andio-CT is recommended in such events, since the visualization of the neck anatomical structures avoids hazardous mishaps.

## Figures and Tables

**Table 1:** Description of the local anatomical problems in our patients.

Anatomical problems	Patients, *n*
Hyper-flexion/impossible neck flexion due to:	11
Musculoskeletal deformation	7
Hallow/C-spine fixation after neck injury or surgery	4
Morbid obesity and low larynx	7
Goiter	3
*Patients with multiple neck anatomical abnormalities among the 21 patients*	*2*

*n*: number.

**Table 2:** List of complications.

Complication	Description and solution	Patients, *n*
Cuff leak	Failure of the cuff to remain inflated at predetermined pressure; increased pressure, increased ventilatory pressure	2

Minor Bleeding	Oozing; frequent dressing changes, direct pressure or suture placement	2

Minor stoma infection	Localized infection; application of topical Antibiotics	1

*n*: number.
